# Comorbid seizure reduction after pallidothalamic tractotomy for movement disorders: Revival of Jinnai’s Forel‐H‐tomy

**DOI:** 10.1002/epi4.12467

**Published:** 2021-02-02

**Authors:** Shiro Horisawa, Satoru Miyao, Tomokatsu Hori, Kotaro Kohara, Takakazu Kawamata, Takaomi Taira

**Affiliations:** ^1^ Department of Neurosurgery Tokyo Women’s Medical University Tokyo Japan; ^2^ Department of Neurosurgery Moriyama Neurological Center Hospital Tokyo Japan

**Keywords:** Forel‐H‐tomy, pallidothalamic tract

## Abstract

Forel‐H‐tomy for intractable epilepsy was introduced by Dennosuke Jinnai in the 1960s. Recently, Forel‐H‐tomy was renamed to “pallidothalamic tractotomy” and revived for the treatment of Parkinson's disease and dystonia. Two of our patients with movement disorders and comorbid epilepsy experienced significant seizure reduction after pallidothalamic tractotomy, demonstrating the efficacy of this method. The first was a 29‐year‐old woman who had temporal lobe epilepsy with focal impaired awareness seizure once every three months and an aura 10‐20 times daily, even with four antiseizure medicines. For the treatment of hand dyskinesia, she underwent left pallidothalamic tractotomy and her right‐hand dyskinesia significantly improved. Fourteen months later, she had experienced no focal impaired awareness seizure and the aura decreased to one to three times per month. The second case was that of a 15‐year‐old boy diagnosed with progressive myoclonic epilepsy, who developed generalized tonic‐clonic seizure, which manifested once every month, despite treatment with five antiseizure medicines. After surgery, myoclonic movements in his right hand slightly improved. A one‐year follow‐up revealed that he had not experienced a generalized tonic‐clonic seizure. The lesion locations in the two cases were close to the vicinity of Jinnai's Forel‐H‐tomy. Forel's field H deserves reconsideration as a treatment target for intractable epilepsy.

## 1 INTRODUCTION

Forel's field is a subthalamic area, which includes three distinct, myelinated fiber bundles, Forel's field H, H1, and H2.^1^ Forel's field is embedded in the basal ganglia‐thalamo‐cortical circuit, which is the principal network involved in the regulation of motor function and complex behavior.^1^ However, the functional distribution of Forel's field remains unclear.^2^ In the 1960s and 1970s, stereotactic ablation of Forel's field H (Forel‐H‐tomy) was performed for the treatment of Parkinson's disease (PD) and cervical dystonia.^3^, ^4^ Almost simultaneously, Jinnai et al reported using Forel‐H‐tomy for treating epilepsy.^5^ Although the ablation of Forel's field was forgotten for several decades, Jeanmonod's group revived Spiegel's Forel‐H‐tomy (renamed to “pallidothalamic tractotomy”) for the treatment of PD using radiofrequency and focused ultrasound ablation.^6^, ^7^

**FIGURE 1:**
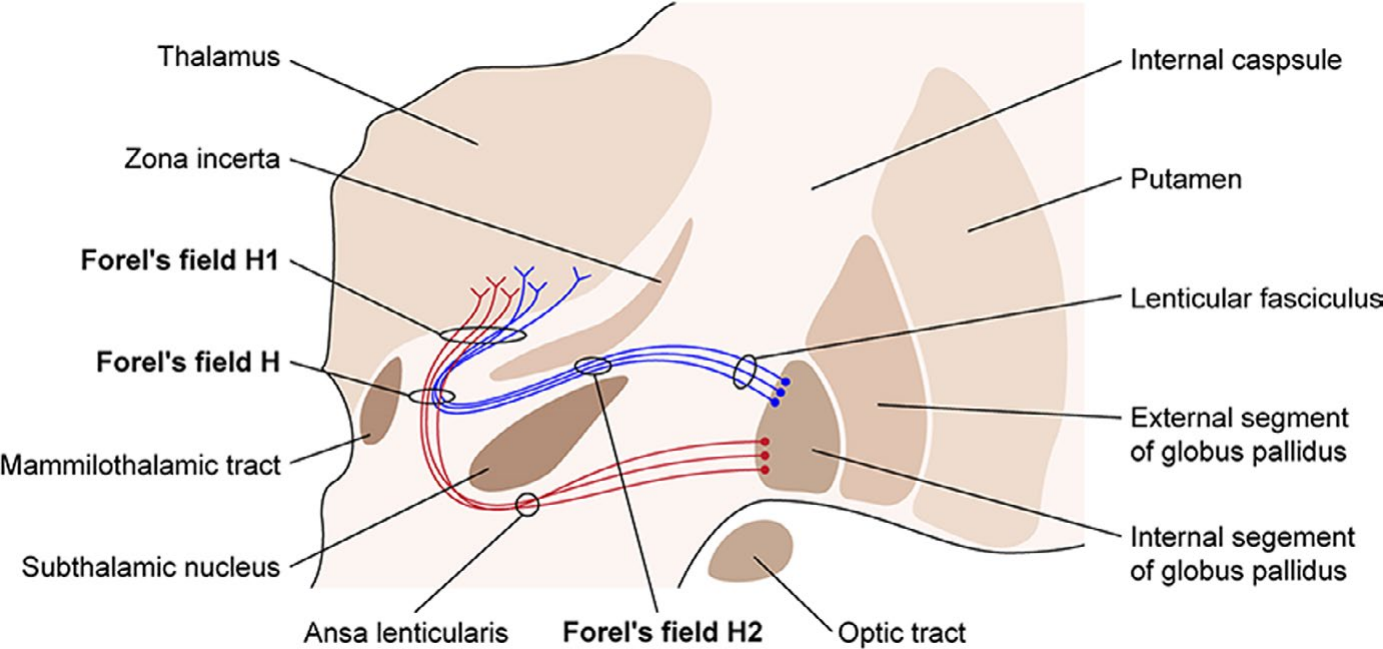
Scheme of the Forel's field H, H1, and H2, and pallidothalamic tract. Pallidothalamic tract consists of ansa lenticularis and lenticular fasciculus. Two fibers merge at Forel's field H and in turn ascend to the thalamus through Forel's field H1. Forel's field H, H1, and H2 and zona incerta are situated close to each other. The lesion of PTT is an area within Forel's field H1

Pallidothalamic tract (PTT) comprises two fiber bundles, the ansa lenticularis and lenticular fasciculus, which connect the globus pallidus internus with the motor thalamus.^2^ The ansa lenticularis courses anteromedially and ventrally around the internal capsule to posterodorsally join the Forel's field H.^2^ The lenticular fasciculus arches over the subthalamic nucleus through Forel's field H2 and subsequently reaches Forel's Field H.^1^ Both fibers course in different ways and merge at Forel's field H, and the merging fiber heads into the motor thalamic nuclei through Forel's field H1 (Figure 1).^1^, ^2^ We have previously reported the efficacy of pallidothalamic tractotomy in treating PD and dystonia.^8^, ^9^ Furthermore, we recently observed significant seizure reduction after pallidothalamic tractotomy in two patients with movement disorders and comorbid epilepsy, which demonstrates the efficacy of this method.

## 2 METHODS

T2‐weighted axial/coronal magnetic resonance imaging (MRI) (1‐mm slice) was used to plan the surgery. The PTT is located above the subthalamic nucleus (STN) and lateral to the mammillothalamic tract (MTT), both of which are clearly visualized as low‐intensity areas on T2‐weighted MRI. According to the location of MTT and STN on the T2‐weighted MRI, we adjusted the ventrocaudal and mediodorsal coordinates for PTT. We set two stereotactic targets to cover the PTT. We used a monopolar radiofrequency probe (1.0‐mm‐diameter tip with an uninsulated length of 4.0 mm) and a Leksell Neuro Generator (Elekta) to induce macrostimulation and coagulation. Coagulation was performed at 70**°**C for 40 seconds at each target. The detailed operative procedure has been described in a previous study.^8^

The seizure frequency was stable for several years before surgery. Seizure frequency was confirmed by each patient's seizure diary. The prescription of antiseizure medicine (ASM) did not change around the surgery.

## 3 RESULTS

### 3.1 Case 1

**FIGURE 2:**
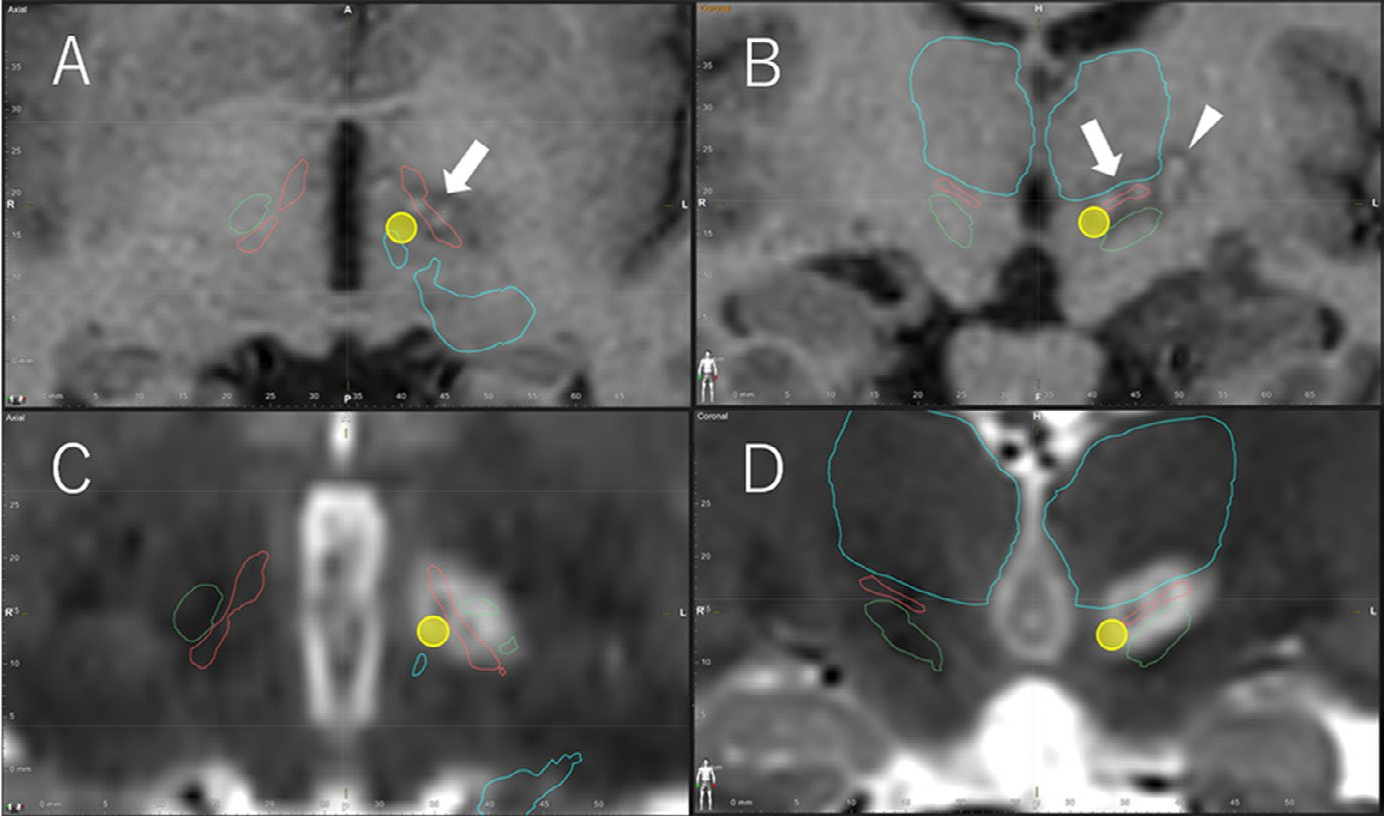
Postoperative MRI images after pallidothalamic tractotomy (Forel‐H‐tomy) in cases 1 (A, B) and 2 (C, D). A and B, Postoperative T1‐weighted axial (A) and coronal (B) MRI images in case 1 showing pallidothalamic lesions (arrow) located between the thalamus and subthalamic nucleus. A ventro‐oral lesion (arrowhead) is located lateral to the pallidothalamic lesions. C and D, Postoperative T2 weighted axial (C) and coronal (D) MRI images in case 2 showing pallidothalamic lesions located between the thalamus and subthalamic nucleus. Yellow circles represent Jinnai's Forel‐H‐tomy locations. The Brainlab Elements Anatomical Mapping Tool was used to delineate anatomical structures surrounding Forel's field H. blue: thalamus; green: subthalamic nucleus; red: zona incerta. MRI, magnetic resonance imaging

A 29‐year‐old right‐handed woman underwent tumor removal of a left medial temporal astrocytoma at the age of nine. She had temporal lobe epilepsy with focal impaired awareness seizure (oroalimentary automatism) once every three months and an aura (a rising sensation in the abdomen) 10‐20 times daily, even when treated with four ASMs (perampanel 4 mg, clobazam 10 mg, clonazepam 1 mg, and valproic acid 400 mg). Interictal surface electroencephalography (EEG) was localized to the left temporal lobe. Bilateral hand dystonia developed at the age of 27 years. For the treatment of right‐hand dystonia, she underwent left pallidothalamic tractotomy and ventro‐oral thalamotomy. The ablative target of the left medial PTT was located 9 mm lateral, 1 mm posterior, and 3 mm inferior to the midpoint of the anterior commissure‐posterior commissure (AC‐PC) line, and the lateral target of the left PTT was located 12 mm lateral, 2 mm posterior, and 1 mm inferior to the midpoint of the AC‐PC (Figure 2A,B). The lesion of the ventro‐oral nucleus was located more inferiorly than we expected, which was mainly on the lateral part of Forel's field H1. Immediately after surgery, her right‐hand dystonia significantly improved (80% improvement in the right arm on the Burke‐Fahn‐Marsden Dystonia Rating Scale‐Movement Scale). Concurrently, the aura was not confirmed for 14 days until discharge. At the 14‐month follow‐up, there had been no focal impaired awareness seizure, and the aura frequency decreased to one to three times per month.

### 3.2 Case 2

A 15‐year‐old right‐handed boy presented with bilateral myoclonic arm movements. At the age of seven, he developed a generalized tonic‐clonic seizure. Initially, the frequency of the seizure was confirmed to be once per year. Around the age of 10 years, the seizure frequency gradually increased to once every three months. At the age of 11 years, myoclonic movements in the body trunk and head were confirmed. Myoclonic movements also developed in the bilateral upper and lower extremities at the age of 13 years. Balance disorder, dysarthria, and cognitive decline progressively developed, and he was diagnosed with progressive myoclonic epilepsy. Interictal EEG showed broad theta waves with intermittent spikes and waves over the bilateral parieto‐occipital region with slowed background activity. Generalized tonic‐clonic seizure manifested once every month, even with the administration of five ASMs (perampanel 10 mg, levetiracetam 1000 mg, clonazepam 1.5 mg, valproic acid 800 mg, and piracetam 21 g). He was referred to our hospital for the surgical treatment of myoclonic movements. The Mini Mental State Examination revealed a score of 22/30. Myoclonic movements in his hands (bilateral) severely interfered with his writing and other daily activities. Due to his right‐handedness, we performed left pallidothalamic tractotomy for right‐hand myoclonic movements. The ablative target was located 9 mm lateral, 1 mm posterior, and 3 mm inferior to the midpoint of the AC‐PC on the medial PTT, and 12 mm lateral, 2 mm posterior, and 1 mm inferior to the midpoint of the AC‐PC on the lateral PTT (Figure 2C,D). After the surgery, the myoclonic movements in his right hand slightly improved without complications (10% improvement on the Unified Myoclonus Rating Scale). At the one‐year follow‐up, the patient had not experienced a generalized tonic‐clonic seizure.

## 4 DISCUSSION

In this study, seizure frequency dramatically decreased in two patients who presented with focal impaired awareness seizure and generalized tonic‐clonic seizure after pallidothalamic tractotomy. In a systematic review of stereotactic lesions for epilepsy treatment, Forel‐H‐tomy achieved an average modified Engel scale score of 1.4 in 32 patients with generalized tonic‐clonic/focal impaired awareness seizure, 1.3 in 20 patients with simple motor seizure, and 2.2 in 11 patients with generalized tonic‐clonic seizure.^10^ Therefore, Forel's field H deserves reconsideration as a treatment target for intractable epilepsy.

Forel‐H‐tomy for intractable epilepsy was introduced by Dennosuke Jinnai in the 1960s. The rationales for Forel‐H‐tomy in treating epilepsy are interrupting the pathway of epileptic discharge (descending impulse) and elevating the seizure threshold based on experimental animal work.^5^ Jelsma et al stated that the reduction of generalized seizures after Forel‐H‐tomy may be related to interruption of pallidothalamic pathways and thalamocortical circuits involving the irritable cortex, thus resulting in blockage of seizure dissemination.^11^ Jinnai et al reported the efficacy of Forel‐H‐tomy in 64 patients with intractable epilepsy.^5^ In well‐placed lesions (unilateral or bilateral), significant seizure reduction (diminished or abolished) was confirmed in generalized convulsions, focal convulsions, and the Lennox‐Gastaut syndrome. Bilateral Forel‐H‐tomy was superior to unilateral Forel‐H‐tomy for seizure control. However, in obvious asymmetrical seizure manifestation cases (symptomatically or electroencephalographically), unilateral Forel‐H‐tomy could achieve good seizure control. Forel‐H‐tomy target was placed 4.5‐5.0 mm lateral to the wall of the third ventricle and 2 mm posterior and 4 mm inferior to the midpoint of the AC‐PC line, which is located posteroinferomedially to our lesions (Figure 1). The Jinnai's target is just anterior to the red nucleus where cerebellothalamic tracts pass through, located 4 mm inferior to the AC‐PC plane according to the Morel's Atlas. The pallidothalamic and cerebellothalamic tracts are situated close together, approximately 2 mm inferior to the AC‐PC plane, suggesting that PTT has a high likelihood of involving both these tracts. Forel‐H‐tomy reduces the degree and frequency of the seizure and/or shortens the seizure duration.^12^, ^13^ Yoshii et al also reported seizure abolishment in 22.2% of patients and significant seizure reduction (frequency and/or degree) in 33.3% of patients after Forel‐H‐tomy at the 1‐year follow‐up.^12^ Jelsma et al reported two patients with generalized tonic‐clonic seizure who were successfully treated using Forel‐H‐tomy with capsulotomy.^11^ In these patients, the seizure frequency dramatically improved from three and two seizures each week to four seizures in 61 months and no generalized seizures in 30 months, respectively. The lesion was performed 11.5 mm lateral to the midline, 1.5 mm below the AC‐PC plane, and with 42% of the AC‐PC distance behind the AC, which is very close to our lesion location.^11^ Andrew et al confirmed that Forel‐H‐tomy with capsulotomy or amygdala lesions was significantly effective for patients with generalized seizure with a focal onset.^14^

Complications reportedly associated with Forel‐H‐tomy include akinetic motor disturbances, speech difficulty, difficulty swallowing, and slight impairment in typing speed.^5^, ^15^ Jinnai et al stated that complications including motor, speech, and swallowing disturbances were confirmed only in cases when lesions were above the AC‐PC plane, which were outside Forel's field H; therefore, precise ablation of Forel's field H was safe even in bilateral ablations.^5^ However, in our experience, patients who underwent unilateral PTT for movement disorders developed mild hypophonia and dysarthria, as well as decreased hand dexterity. Considering the fact that these complications are associated with unilateral surgery, patients who undergo bilateral Forel‐H‐tomy may develop serious speech and motor disturbances. To avoid possible complications associated with lesions, deep brain stimulation (DBS) can be a treatment option. DBS disrupts abnormal information flow through the stimulation site.^16^ Therefore, DBS provides similar therapeutic effects to lesioning surgery. The disruption of pathological information flow associated with movement disorders or epilepsy at PTT may achieve symptomatic improvement. Although no reported cases of DBS of Forel's field H for epilepsy exist, Elhadd et al recently reported a case of dystonic tremor with generalized epilepsy (juvenile myoclonic epilepsy) treated with DBS targeting the ventral intermediate nucleus and zona incerta (ZI).^17^ The stimulation not only improved the dystonic tremor but also dramatically decreased seizure frequency from two to three seizures each week before surgery to two seizures in three years after surgery. Co‐stimulation of Forel's field H1 or H2 may be responsible for the dramatic seizure reduction.^14^

One of the limitations in this study is the anatomical location of Forel's field H1‐tomy. Our method of PTT is intended to make a lesion within Forel's field H1 as disrupting the basal ganglia‐thalamo‐cortical circuit is necessary to improve dystonic movement disorders. However, Forel's field H, H1, and H2, as well as ZI, are situated close to each other, and it is impossible to selectively make a lesion within Forel's field H1. Our lesions on the postoperative MRI include not only Forel's field H1 but also Forel's field H2 and ZI. Therefore, we could not precisely distinguish the acquired results from Forel's field H1 or H2, or the lesion within ZI. Another limitation is a lack of postoperative EEG collection.

In conclusion, Forel's field H, H1, and H2 deserve reconsideration as treatment targets for intractable epilepsy.

## CONFLICTS OF INTEREST

None of the authors have any conflicts of interest to disclose. We confirm that we have read the journal's position on issues involved in ethical publication and affirm that this report is consistent with those guidelines.
